# Mining rich health data from Canadian physician claims: features and face validity

**DOI:** 10.1186/1756-0500-7-682

**Published:** 2014-10-01

**Authors:** Ceara Tess Cunningham, Pin Cai, David Topps, Lawrence W Svenson, Nathalie Jetté, Hude Quan

**Affiliations:** Department of Community Health Sciences, University of Calgary, 3280 Hospital Dr. NW, Calgary, Alberta T2N 4Z6 Canada; Reporting and Data Management, Alberta Health Services, Calgary, Alberta Canada; Department of Family Medicine, University of Calgary, Calgary, Alberta Canada; Surveillance and Assessment Branch, Alberta Health, Edmonton, Alberta Canada; School of Public Health, University of Alberta, Edmonton, Alberta Canada

**Keywords:** Administrative data, International classification of diseases, Quality, Validity

## Abstract

**Background:**

Physician claims data are one of the largest sources of coded health information unique to Canada. There is skepticism from data users about the quality of this data. This study investigated features of diagnostic codes used in the Alberta physician claims database.

**Methods:**

Alberta physician claims from January 1 to March 31, 2011 are analyzed. Claims contain coded diagnoses using the International Classification of Diseases, 9th revision (ICD-9), procedures, physician specialty and service-fee type. Descriptive statistics examined the diversity and frequency of unique ICD-9 diagnostic codes used and the level of code extension (e.g. 3- or 4-digit coding).

**Results:**

A total of 7,441,005 claims by 6,601 physicians were analyzed. The average number of claims per physician was 1,079, with ranges between 1,330 for family medicine, 690 for internal medicine, 722 for surgery, 516 for pediatrics and 409 for neurology. Family physicians used an average of 121 diagnostic codes, internal medicine physicians 32, surgery 36, pediatrics 46 and neurology 27. Overall, 43.5% of claims had a more detailed diagnosis (ICD code with >3 digits). Physicians on a fee-for-service plan submitted 1,184 claims and used 88 unique diagnosis codes on average compared to 438 claims and 44 unique diagnosis codes from physicians on an alternative payment plan (APP).

**Conclusions:**

Face validity of diagnosis coded in physician claims is substantially high and the features of diagnosis codes seem to reasonably reflect the clinical specialty. Physicians submit a diverse array of ICD 9 diagnostic codes and nearly half of the ICD-9 diagnostic codes examined were more detailed than required (i.e. ICD code with >3 digits). Finally, guidelines and policies should be explored to assess the submission of shadow billings for physicians on APPs.

## Background

Canadian fee-for-service physicians must submit claims to their provincial government health care insurance program in order to be remunerated [1, 2]. Even though various forms of physician reimbursement models exist in Canada, the majority of physicians need to submit claims outlining their clinical services, based on a set of billing codes established by the payer [3, 4]. The provincial physician claims database contains demographic and clinical information, such as a patient’s diagnosis at the time of a visit. Consequently, physician billing databases capture relatively complete and comprehensive information on inpatient and outpatient physician services for all specialties [5], representing a rich and detailed source of health information that is unique to Canada [6].

In Canada, every citizen is covered by universal health. Canadians are covered whether or not they can afford health care. As a result, unlike in the United States (US) where universal health care coverage does not exist, the majority of contacts with the health care system in Canada (95%) are captured in administrative health databases such as the provincial physician claims databases. Despite the US’s publicly funded health care program (e.g. Medicare and Medicaid) that help provide care to more vulnerable populations (i.e. disabled individuals, elderly), the completeness of the medical information derived from these US data sources are not generalizable to the whole US population.

Physician billing claims can be linked to other administrative health databases (such as inpatient discharge abstract data and vital statistics) to capture patients’ clinical and outcome information within and outside of hospitals. These linked data are often used for chronic disease surveillance to examine health service utilization and for outcome research [7–11]. The validity of physician claims is questionable. Skepticism about billing claims’ data quality comes directly from the billing processes and the potential errors that may occur prior to and during their submission [12]. Payment staff in some provinces check missing values and clinical logics in the submitted claims [13] but rarely verify the accuracy of coded diagnoses. Only the procedure code (e.g. major assessment visit), which is linked to the level of reimbursement, is carefully checked [14]. Anecdotal evidence indicates physicians may be less concerned with the accuracy of the diagnosis and use a limited number or less detailed International Classification of Disease (ICD) codes to code diagnoses. Little research exists to refute or support these claims. Lix et al. [15] described the major features of physician services databases in Canada. Other Canadian researchers have validated physician claims in recording individual conditions [16–19]. Overall however, the quality of the data has not been assessed and physicians billing claims still remains a source that has not been fully explored or validated [18, 20].

Validity of physician claims has been further questioned because recently, different payment models for physicians have been introduced across Canada. Alternative payment plan (APP) physicians are paid a fixed amount of money with additional remuneration components, rather than being paid for each service they provided. APP physicians do not have to submit a claim in order to be paid for each service provided. However, some provinces including Alberta request that APP physicians to submit claims (called shadow billing) primarily to ensure that physicians are providing the services indicated in their contracts. Because FFS physicians must submit a claim to be paid, it has been assumed that this payment system provides relatively complete and accurate estimates of medical service provision. Additionally the quality and accuracy of the FFS physicians’ claims is thought to be fairly accurate. However, since APP models do not necessarily require physicians to submit billing claim information, their submissions are not verified by insurance plans as thoroughly as FFS claims. This gap within payment models may create potential inequities in the quality of claims and result in data erosion, such as decreased frequency of claims submitted and missing clinical information.

Currently, over 20% of Canada’s 55,000 physicians receive some payments for clinical care from APPs [21]. With the growing rise in APP implementation, the quality of data gathered from physicians’ billings has come into question due to the suspected non-submission or poor accuracy of clinical information these APP physicians may be submitting [22, 23]. The usefulness of physician claims, for research, surveillance, healthcare planning and decision-making depends on accurate data submission.

Given this background, the purpose of this study was to explore the features and face validity of the physician claims database in a large Canadian province; specifically we assessed the diversity and frequency of utilization of unique diagnosis codes and the extension of diagnosis codes. A paper such as this is critical in establishing the foundation for future use of physician claims data for research in Canada.

## Methods

### Study population

The population of the province of Alberta is about 3.8 million [24]. We identified all claims between January 1, and March 31, 2011 from the Alberta provincial physician claims database. The claims database contains unique patient identifiers, unique physician identifiers, up to three ICD-9 diagnostic codes, one procedure code (using the Canadian Classification of Procedures), provider specialty, fee for services provider and functional centre type (i.e. where the service was provided). APP physicians in Alberta are expected to submit billing claims (shadow bill claims), even if they are not necessary for remuneration. As a result, there is a flag or billing indicator in the physician claims database to indicate which of the submissions are from APP physicians.

**Figure 1:**
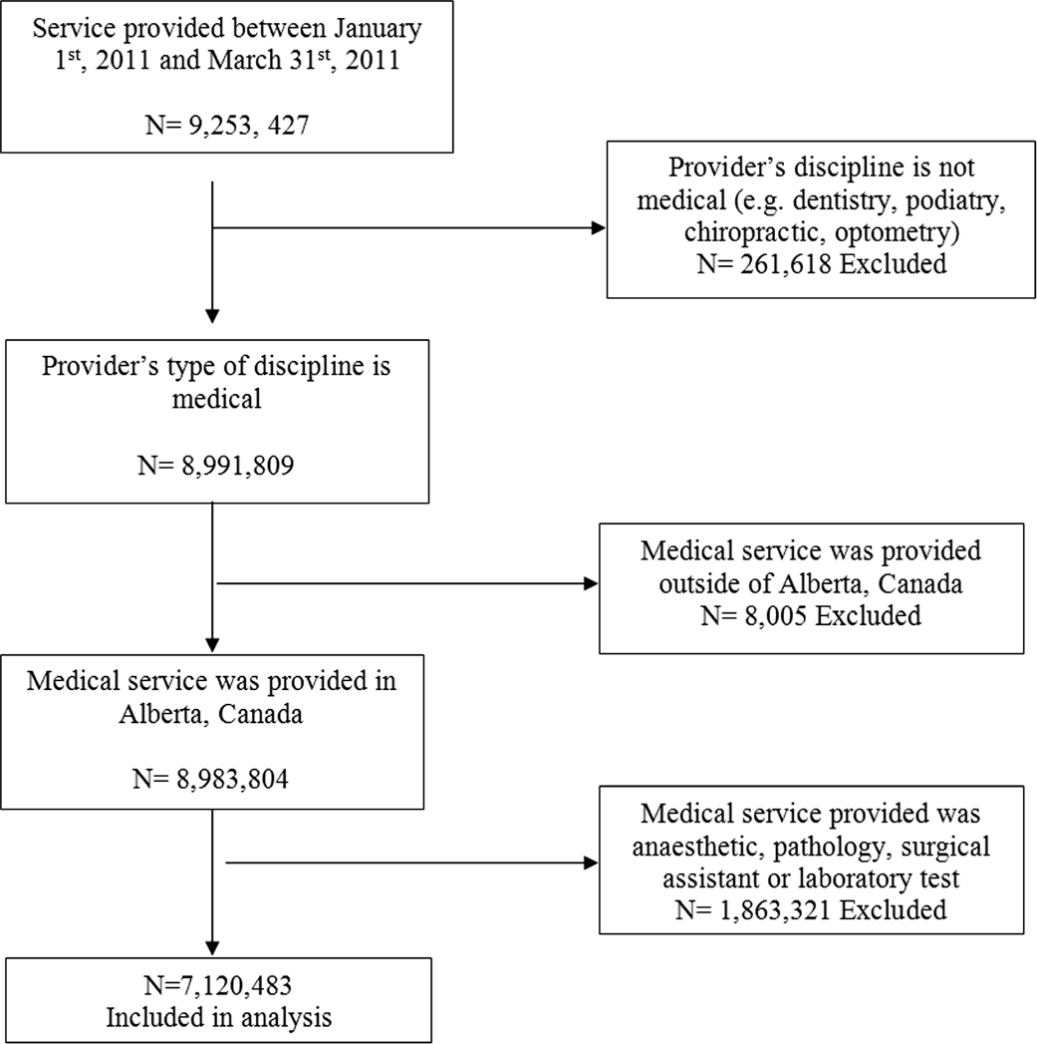
**Sample selection of claims records.** Analysis was limited to a three-month period; still capturing 7,441,005 eligible claims for this study. Claims were excluded if the health service provided was an x-ray, anaesthetic service, surgical assistant service or laboratory test or if the provider’s specialty was pathology or anaesthesiology.

Given the large amount of claims in the database, we limited our analysis to a three-month period; still capturing 7,441,005 eligible claims for this study (see Figure 1). Claims were excluded if the health service provided was an x-ray, anaesthetic service, surgical assistant service or laboratory test or if the provider’s specialty was pathology or anaesthesiology. These claims were excluded as these medical services are considered diagnostic and are processed differently than physician billing claims.

### Analysis

It is possible for a physician to submit multiple claims for an individual patient on the same day depending on the services provided. Thus our analysis unit was an individual patient’s visit, which was defined as a visit that happened on the same day, for the same patient, by the same provider, and at the same service delivery site. A distinctive diagnosis code was defined by the first three digits of an ICD-9 diagnosis code since it is the minimum requirement for diagnostic detail. More expanded ICD-9 codes with 4th or 5th digit are optional. Descriptive statistics were used to report the number of claims, visits and distinctive diagnosis codes used per physician. The analysis was stratified by specialty and payment program (fee-for-services vs. APP). In Canada, primary care (family medicine) physicians are the forefront of Canadian health care and provide basic medical check, treatments and preventative care. People, regardless of age, may see a primary care physician, who may choose to refer the patient to a specialist physician (including pediatrics or internal medicine) for further evaluation. In Canada, pediatricians and internists are not considered primary care physicians as they are in the US and thus they were analyzed separately from the family medicine physicians. All statistical analyses were conducted using SAS, Version 9.3 TS1M1 (SAS Institute Inc, Cary, NC, USA). This study is based in part on data provided by Alberta Health. The interpretation and conclusions contained herein are those of the authors and do not necessarily represent the views of the Government of Alberta. Neither the Government nor Alberta Health express any opinion in relation to this study. Ethics approval for this study was granted by the University of Calgary Conjoint Health Research Ethics Board.

## Results

A total of 7,441,005 claims were submitted by 6,601 physicians in the three month study period (see Table 1).

**Table 1:** **Physician specialty and average number of claims per physician, 3-month period, January-March 2011**

Specialty	Number of Physicians	Number of claims				
		N	Mean	Median	IQR25	IQR75
Total	6,601	7,120,483	1,079	865	359	1,493
Family medicine	3,449	4,585,486	1,330	1,161	600	1,804
Internal medicine	795	548,523	690	546	285	934
Neurology	106	43,335	409	332	226	495
Obstetrics and Gynecology	176	195,078	1,108	1,106	504	1,455
Pediatrics	382	196,968	516	330	135	689
Psychiatry	395	358,956	909	691	295	1,344
Radiology	261	34,164	131	68	22	177
Surgery	462	333,774	722	690	381	952
Others	575	824,199	1,433	1,078	591	1,679

The average number of claims submitted per physician was 1,079, with a high ranges between 1,330 for family medicine, 690 for internal medicine and 722 for surgery and lower ranges such as 516 for pediatrics and 409 for neurology. The average number of unique diagnosis codes submitted for all specialties was 82 (see Table 2). Family physicians used an average of 121 diagnostic codes, internal medicine physicians 32, surgery 36, pediatrics 46 and neurology 27. The mean and median number of diagnostic codes was not included in analysis as although physicians can provide up to 3 diagnosis codes per claim, approximately 94% only provide one diagnosis per claim. Overall, 43.5% of claims contained a more detailed ICD-9 code (>3 digits), with this proportion being highest among pediatricians at 58.4% and lowest for family medicine at 40%.

**Table 2:** Average number of diagnosis and procedure codes per physician and ICD* precision, 3-month period, January-March 2011

Specialty	ICD diagnosis codes*			Procedure codes			Claims with ICD 4th or 5th digit	
	Mean	Median	IQR ^†^	Mean	Median	IQR	Number	%
Overall	82	60	21-134	21	16	3 -29	3,239,628	43.5%
Family medicine	121	122	70-171	20	17	10-27	1,907,011	40.0%
Internal medicine	32	25	16-43	15	14	9-20	326,583	55.2%
Neurology	27	24	16-34	10	10	7-12	25,224	53.5%
Obstetrics and Gynecology	37	37	20-52	40	44	26-55	114,016	58.2%
Pediatrics	46	37	15-73	12	11	7-16	121,377	58.4%
Psychiatry	10	10	6-13	10	10	6-15	147,100	37.7%
Radiology	12	8	5-14	12	8	5-15	20,994	57.5%
Surgery	36	33	23-46	44	46	32-59	199,631	57.6%
Others	76	55	20-134	29	30	15-41	377,692	44.3%

*ICD: International Classification of Disease, precision increases with number of digits including up to 5 digits. Claims with ICD 4th to 5th digit is optional for claims submission.

^†^Inter-quartile range.

The number of unique ICD-9 codes submitted in billing claims varied by payment program (see Table 3). Fee-for-service physicians submitted more diverse ICD codes than APP physicians (88 vs. 44 unique codes respectively). Claims submission on average was lower for APP physicians than fee-for-service physician (438 vs. 1,184). The most frequent reasons for each visit by specialty are reported in the appendices.

**Table 3:** **Average number of ICD diagnosis codes per physician in physician claims by payment program, 3-month period, January-March 2011**

Specialty		Fee-for-service				Alternative payment program		
	Average claims	Mean	Median	IQR*	Average claims	Mean	Median	IQR
Overall	1,184	88	73	22-143	438	44	28	17-54
Family medicine	1,382	124	124	74-173	586	88	75	47-129
Internal medicine	900	37	29	17-51	431	26	22	15-35
Neurology	599	33	34	14-50	323	23	23	16-31
Obstetrics and Gynecology	1,145	38	37	20-51	436	32	16	15-52
Pediatrics	681	54	52	12-86	296	35	25	16-44
Psychiatry	917	11	10	6-13	394	11	9	5-19
Radiology	131	12	8	5-14	N/A		N/A	
Surgery	756	36	34	23-47	383	28	25	20-32
Others	1,531	79	72	21-137	488	42	23	17-48

*Inter-quartile range.

## Discussion

We analyzed Alberta wide physician claims data to understand the face validity of clinical information recorded in this physician claims database. This study highlighted that: 1) Physicians submitted a diverse array of ICD 9 diagnostic codes; 2) Nearly half of the ICD-9 diagnostic codes examined were more detailed than required; for example, the ICD-9 code 250 is coded as diabetes (3-digit minimum) and this can be further specified by using the 4-digit coding such as 250.3, diabetes with renal manifestations; 3) ICD-9 diagnostic codes used were more diverse for fee-for-services physicians than APP physicians and 4) The most frequently submitted reason for visit was consistent with the physician specialty. Thus, our findings indicate physicians in Alberta use a broad and diverse structure of diagnosis coding across specialties.

While certain physicians or specialists may use specific or common codes familiar to their practice, it appears the patterns of use of ICD-9 codes reflect their clinical practice. For example, family doctors were the least likely to use 4-digit or 5-digit coding. There may be a number of reasons for this. Codes used by family doctors may be initially investigative, and therefore not as detailed or specific as specialists’ codes. Also, family doctors often provide referrals to other specialists limiting the specificity of the code used. This may also be a reflection of the limitations of the ICD-9 coding system for primary care. Katz et al. [25] reported that the use of the ICD-9 coding system was not sensitive to the breadth and complexity of primary care encounters (such as ICD-9 codes) contributing to a limited amount of codes used by family physicians. Our findings also indicate specialist physicians (e.g. pediatrics, internal medicine, surgery) are more likely to use 4-digit or 5-digit coding. These physicians are not just using a few overarching ICD-9 codes or unspecific codes. These results support the relatively high face validity of Canadian physician claims’ database. Physician claims data in Canada are therefore highly valuable for research, surveillance and healthcare planning purposes. However, further validation work is still needed for specific purposes.

Previous studies support our findings that physician claims have a great potential to be used for chronic disease surveillance. Because chronic diseases are commonly managed in outpatient clinics, surveillance of these conditions can be conducted in a timely and efficient manner through the use of physician claims databases. For example, Robitaille et al. [10] reported age and sex adjusted hypertension incidence and prevalence in Canada using physician claims and hospital discharge abstract data. Before utilizing these claims for epidemiological purposes, they had been validated. Quan et al. [17] linked 3362 general practitioner /family physician charts with physician claims in the provinces of Alberta and British Columbia. The diagnostic accuracy of hypertension coding in physician claims data was as follows: sensitivity 73%, specificity 95%, positive predictive value (PPV) 82%, and negative predictive value (NPV) 91%.

Other chronic conditions have been validated in administrative data. For example, Hux et al. [26] reviewed 3317 primary care physician charts and linked these data with the Ontario physician claims and hospital discharge abstract data. The reported validity of diabetes (defined as two physician claims or one hospitalization with a diabetes ICD code) was high, with a sensitivity 86%, specificity 97.1%, PPV 80%, and NPV 99%. Reid et al. [27] reported that physician claims data had a sensitivity of 88%, specificity 92%, PPV 89% and NPV 92% in recording epilepsy compared with neurologist chart data. Ronksley et al. [28] reported a case-definition algorithm employing two physician claims or one hospitalization within a two year period with a sensitivity of 19%, specificity of 97%, PPV of 60% and NPV of 85% for detecting chronic kidney disease compared to the reference standard of estimated glomerular filtration rate. Systematic reviews for rheumatic diseases, heart failure and neurological conditions also demonstrate variation in the validity of ICD coding in administrative data across conditions and studies [29–31].

In the US as in many countries, ICD coded databases are used for many purposes including analysis of morbidity and mortality trends. The use of ICD coding for reimbursement represents an important aspect of health care operations and administration in many countries, particularly in the US [12]. For example, the Veterans Health Administration uses ICD codes to set capitation rates and allocate resources to medical centers caring for its beneficiaries. When Medicare’s Prospective Payment System (PPS) was enacted, diagnosis-related groups (DRGs) based on ICD codes emerged as the basis for hospital reimbursement for acute-care stays of Medicare beneficiaries [12, 32]. Similar to Canadian administrative data, these types of coded health data are enormously beneficial for disease surveillance, budgetary allocations and health services research. However, US ICD data are mainly from hospitals and lack national information of outpatients. Surveillance based on hospital ICD data is susceptible to sources of selection bias and systematic error. Missing information on the complexity, location and severity of certain conditions can lead to underestimates of certain chronic diseases and result in confounding in study findings [33].

In Canada, we found the face validity varied by physician payment program. Physicians on an APP are submitting approximately half the claims on average and used fewer distinctive ICD-9 diagnostic codes than physicians on a fee-for-service plan. Possible reasons for this are that some APP physicians may not be submitting some claims, may pay less attention to ICD-9 coding or both. Physicians on an APP are generally required to submit claims for services provided, called “shadow bills”, for administrative purposes [34]. Unfortunately, APPs do not generally provide financial incentives for physicians to submit claims for all their services (e.g. they are not compensated for the time spent billing).

Advantages of mandatory submission of APP claims is that such a mechanism could potentially decrease incomplete and inaccurate billing submissions. This in turn would result in an increase in ability to effectively track health service volume and utilization and as a result better estimate the burden of diseases. Furthermore, mandatory submission of APP claims would enhance the usefulness and overall validity of administrative databases. Unfortunately, mandatory billing incentives could decrease physicians’ overall satisfaction with APP contracts and could decrease physicians’ willingness to participate in APP programs.

Some provinces in Canada are realizing the potential for data loss and have begun to generate policies to promote shadow-billing submissions. Alberta is one of several Canadian provinces that require APP physicians to submit shadow bills to account for the services they provide. In addition, many provincial APP programs based at teaching hospitals utilize incentive-based programs to motivate physicians to submit billings. For example in some divisions or departments APP physicians who do not submit the recommended quota of shadow billings based on their expected patient workload annually face a potential withholding (e.g. 15%) of their yearly earnings [35]. Physicians are now being monitored and given multiple warnings and are able to review their billings and can resubmit to fulfill this required mandate within their contracts. This type of policy is important and demonstrates that provincial governments and health agencies are beginning to realize the possible risk of data erosion with the switch to APPs and are implementing policies to adjust for the under-submissions of shadow bills.

According to more recent (2010) data from the Canadian Institute for Health Information (CIHI), the majority of physicians receive payments from both fee-for-services and APP programs and a great amount of diversity exists across provinces [3]. Unfortunately, reporting systems for shadow billing in the provinces of Alberta, British Columbia, and Saskatchewan are not standardized [3]. This could lead to large differences in the completeness and accuracy of data within physician claims databases across provinces. The erosion of data due to shadow billing is a growing concern but has not been adequately explored. Canadian policy makers advocate for APP programs but little attention has been paid to sustain or improve the quality of claims data for APP physicians [36].

However, we cannot confirm from our study whether data erosion is indeed occurring when physician switch from fee for service to APPs. For example, some may argue that it is not surprising that APP physicians are submitting less billing claims and use less ICD codes than fee for service physicians because in Alberta, APP physicians are academic physicians. Academic physicians not only provide clinical care, but are also required to teach, do clinical research and/or be involved in a variety of other academic activities. Thus, only a small proportion of academic physicians provide clinical care more than three days per week. These academic physicians are also generally more likely to be specialized or even subspecialized, thus seeing a smaller variety of acute and chronic conditions, which may explain why they are submitting fewer codes.

This study has several limitations. First, our analysis is limited to Albertan physician claims data and therefore may not be generalizable to other Canadian provinces. Second, we did not estimate non-submissions from APP physicians and validate submitted clinical information. Future studies are needed to address these two critical questions. Third, we did not evaluate the impact of shadow billing on disease burden estimate and outcome research. Fourth, in this study, we described claim patterns between APP and FFS physicians. The impact of APPs on disease surveillance (such as frequency of various diagnoses, upper respiratory infection, HIV, various cancers) should be considered and examined in future studies. Finally, we did not have a “gold standard” (e.g. chart review, physician report/survey) to validate the claims’ data used in our study.

## Conclusions

The findings from this study offer valuable insight into one of the largest and richest sources of Canadian administrative health data. Although data loss may be occurring due to APPs, the data has great value for health services research, surveillance and healthcare policy development. There is a need to ensure new policies to protect the collection of data on the encounters between physicians and their patients as this evidence can be used to support multiple goals that inform treatment and management of patients through the management of the system as a whole.

## Abbreviations

APP: Alternative payment plan
CIHI: Canadian Institute for Health Information
FFS: Fee-for-service plan
ICD: International classification of diseases.
